# Are Geographical “Cold Spots” of Male Circumcision Driving Differential HIV Dynamics in Tanzania?

**DOI:** 10.3389/fpubh.2015.00218

**Published:** 2015-09-29

**Authors:** Diego F. Cuadros, Adam J. Branscum, F. DeWolfe Miller, Susanne F. Awad, Laith J. Abu-Raddad

**Affiliations:** ^1^Infectious Disease Epidemiology Group, Weill Cornell Medical College in Qatar, Qatar Foundation, Cornell University, Doha, Qatar; ^2^Department of Healthcare Policy and Research, Weill Cornell Medical College, Cornell University, Ithaca, NY, USA; ^3^College of Public Health and Human Sciences, Oregon State University, Corvallis, OR, USA; ^4^Department of Tropical Medicine and Medical Microbiology and Pharmacology, University of Hawaii, Honolulu, HI, USA; ^5^Vaccine and Infectious Disease Division, Fred Hutchinson Cancer Research Center, Seattle, WA, USA

**Keywords:** male circumcision, HIV, medical geography, Tanzania, spatial analysis

## Abstract

**Background:**

Growing evidence suggests significant geographic clustering of male circumcision (MC) in Tanzania. The impact of spatial heterogeneity of MC prevalence on HIV transmission dynamics in this country is not well documented. The aim of this study was to assess the spatial association between MC and HIV infection in Tanzania.

**Methods:**

Data from three Demographic and Health Survey rounds conducted in Tanzania were analyzed to identify spatial associations between MC and HIV using bivariate local indicators of spatial association (LISA). Spatial clusters with low MC prevalence (MC cold spots) were identified using scan statistics. HIV incidence rates for males and females within and outside the MC cold spots were calculated.

**Results:**

Local indicators of spatial association analysis indicated a significant association between MC and HIV in the northern and southwestern regions of Tanzania. Scan statistics identified two MC cold spots in the same locations. Males located outside the MC cold spots had the lowest HIV incidence rate at 0.28 per 100 person-years at risk (pyar). HIV incidence in females located outside the MC cold spots increased from 0.40/100 pyar during 2004–2008 to 0.68/100 pyar in 2008–2012.

**Conclusion:**

Our study provides evidence for a geographic association between MC and HIV in Tanzania. MC could be one of the key factors driving the geographical distribution of the HIV epidemic in the country. Furthermore, in areas where most males are circumcised, the HIV infection burden could be concentrating in the female population. Therefore, along with the voluntary medical MC program, efforts targeting the female population should also be considered.

## Introduction

Three randomized controlled trials have suggested that male circumcision (MC) lowers the risk of men acquiring HIV through female-to-male transmission by about 60% (1). Based on this evidence, the World Health Organization (WHO) and the Joint United Nations Programme on HIV/AIDS (UNAIDS) recommended scaling up voluntary medical male circumcision (VMMC) as an HIV prevention strategy in 14 countries (including Tanzania) with high HIV prevalence and low MC rates (2, 3).

The prevalence of MC in Tanzania has substantial regional variation (4, 5). MC prevalence in the eastern part of the country is as high as 95%. In contrast, only about 26% of the males in the northwestern region of Tanzania are circumcised (4, 5). HIV prevalence in the country also exhibits stark geographical variation (6–8). Most HIV infections are concentrated in the western part of the country, whereas HIV prevalence remains low in eastern Tanzania (6).

In 2009, the government of Tanzania started implementing VMMC as a prevention strategy (9). In light of the regional variation of MC prevalence in Tanzania, resources have been concentrated in locations where MC prevalence is low. Eight regions were the target for the VMMC scale-up program, including Iringa, Kagera, Mara, Mbeya, Mwanza, Rukwa, Shinyanga, and Tabora. While progress toward increased MC coverage has been reported in these areas (9, 10), the overall impact of spatial heterogeneity of MC on the HIV dynamics in Tanzania is not well documented.

Understanding the patterns in which HIV epidemics evolve in areas with low and high MC prevalence, as well as the impact of VMMC on HIV incidence and prevalence is critical for targeting resources toward populations at higher risk of HIV acquisition. Therefore, we assessed the spatiotemporal association between MC and HIV prevalence in Tanzania using spatial epidemiology techniques applied to data from three rounds of the Demographic and Health Survey.

## Materials and Methods

### Data sources

**Figure 1:**
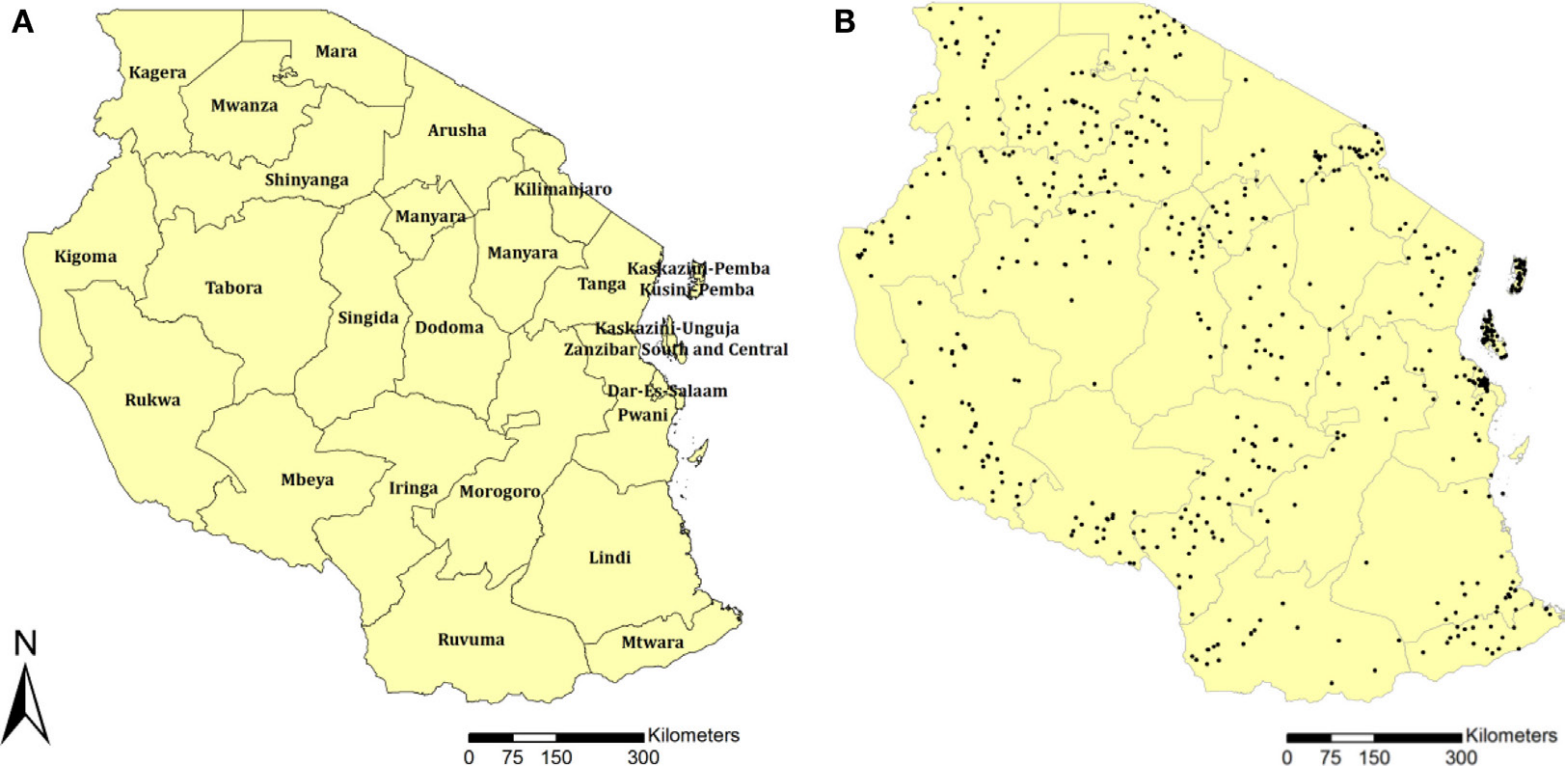
**Tanzania districts (A), and sample locations for the TDHS 2011–12 (B)**.

The main source of data was the Tanzania Demographic and Health Surveys (TDHS) conducted during 2003–04 (11), 2007–08 (12), and 2011–12 (13). These cross-sectional surveys collected data on an HIV serological biomarker and the geographical coordinates of each surveyed location. The three surveys enrolled subjects by using a two-stage sampling procedure to select households. The first stage involved selecting sample locations. A total of 345 TDHS locations (87 in urban and 258 rural areas) were selected in 2003–04, 475 (110 in urban and 365 in rural areas) were selected in 2007–08, and 538 (134 in urban and 449 in rural areas) in 2011–12. The global positioning system was used to identify and record the geographical coordinates of each TDHS sample location. Figure 1B illustrates the geographical position of each sample location for the TDHS 2011–12. Each black dot represents a sample location in which household data were collected. Men and women in the selected households from each sample location aged 15–49 years were eligible for the surveys. The final sample population for data analysis consisted of 5659 men and 6863 women in 2003–04, 6975 men and 9343 women in 2007–08, and 8116 men and 10,693 women in 2011–2012.

Anonymous HIV testing was performed with the informed consent of all sampled men and women. HIV serostatus was determined by testing with the enzyme-linked immunosorbent assay (ELISA) Vironostika Uniform 2 Ag/AB. All samples that tested positive and a random sample of 10% of samples that tested negative on the first ELISA test were retested with a second ELISA, the Enzygnost^®^ HIV Integral II assay (Siemens). Positive samples on both tests were classified as positive. If the first and second tests were discordant, the two ELISAs were repeated; if the results remained discordant, a confirmatory test, the HIV 2.2 western blot (DiaSorin), was administered (13). The binary answer to the question: “*is the respondent circumcised*” was used as the measure of MC status. Further details related to the TDHS methodology, study design, and data can be found elsewhere (11–13).

### Spatial association between male circumcision and HIV

Spatial associations between MC and HIV were investigated using data from the most recent TDHS (2011–12). The prevalence of HIV and the prevalence of MC were estimated at each TDHS sample location, which are illustrated in Figure 1B as black dots. The estimated HIV prevalence (*p*_HIV_) at sample location *i* was defined to be *p*_HIVi_ = *H*_HIVi_/*N*_i_, where *H*_HIVi_ denotes the number of sampled people from location *i* who were HIV seropositive and *N*_i_ denotes the total number of sampled individuals at location *i*. The estimated MC prevalence (*p*_MC_) at sample location *i* was defined to be *p*_MCi_ = *H*_MCi_/*M*_i_, where *H*_MCi_ denotes the number of circumcised males in the sample taken at location *i* and *M*_i_ denotes the total number of males in the sample taken at location *i*. We used the ArcGIS software to generate continuous surface maps of HIV seroprevalence and MC prevalence using kriging interpolation (14, 15). Each map was generated in raster format of one kilometer resolution. Afterward, these two raster maps were then analyzed in the GeoDa environment (16) to assess the spatial correlation between HIV seroprevalence and MC prevalence. The spatial correlations were identified using bivariate local indicators of spatial association (LISA). The bivariate LISA statistics identified significant spatial clustering based on the degree of linear association between HIV prevalence at a given location and the average MC prevalence at neighboring locations (17). Maps were generated to illustrate the locations with statistically significant associations along with the type of spatial association between HIV and MC prevalence (i.e., high–high, low–low, low–high, and high–low).

### Spatial cold spots of male circumcision

Data from the latest TDHS (2011–12) were used to identify spatial clusters with low numbers of circumcised males (MC cold spots) in Tanzania using Kulldorff spatial scan statistics (18–20). For this analysis we used data from only the males who were sampled at each survey location (Figure 1B).

Spatial scan statistics were used for cluster detection to locate areas that have higher or lower numbers of cases (in this study, low numbers of circumcised males) than expected under spatial randomness. The statistical significance of identified clusters is determined by gradually transversing the study region with circular windows (18). The circular windows vary continuously in both location and radius. The radius ranges from slightly above 0 km up to a fixed maximum value, thus creating and testing a large number of distinct potential clusters throughout the study region. A maximum radius that covered 50% of the study region was used. Each potential cluster was tested using a likelihood ratio test to determine the statistical significance against the null hypothesis of spatial randomness (20). Clusters with a *p*-value <0.05, calculated using Monte Carlo simulations, were identified as statistically significant MC cold spots.

Sample locations (black dots in Figure 1B) within the MC cold spots and outside these areas were identified, and the females and males enrolled in the study at each sample location were placed into two disjoint subgroups, individuals located within the MC cold spots and individuals located outside these areas. Using data from the male and the female study population jointly and separately, the fractions of the total population and the male and female subpopulations within and outside the identified MC cold spots were estimated separately using the 2003–04, 2007–08, and 2011–12 TDHS survey data. Total HIV prevalence, HIV prevalence in males and females, and MC prevalence were also estimated within and outside the MC cold spots for the three TDHS rounds. Statistically significant differences between HIV prevalence among males and females within and outside the MC cold spots for each round, and between MC prevalence within and outside the MC cold spots in the first (2004) and last (2012) rounds were identified using chi-square tests. The adjusted relative risk (RR) of HIV infection comparing females to males was estimated both within and outside the MC cold spots for all three rounds. The RR calculations were adjusted for variables known to be associated with HIV in Tanzania (21, 22), such as age, place of residence, wealth index, marital status, education, and presence of genital ulcerations.

### Geographical distribution of HIV incidence rate

HIV incidence rates for males and females were calculated within and outside the MC cold spots during two time periods (2004–2008 and 2008–2012) using a methodology developed by Hallett et al. (23). Briefly, this method is used to estimate the HIV incidence using two cross-sectional surveys by assuming that the observed changes in HIV seroprevalence between the two cross-sectional prevalence measures can be decomposed into contributions due to incident infections and mortality. Individuals of age *a* years in the first survey correspond to individuals aged *a* + γ in the second survey, where γ is the year interval between surveys. Therefore, the change in HIV prevalence among these two age groups could be attributed to the combination of new HIV infections and AIDS mortality. To estimate HIV incidence rate for a given age group, the method uses a value for the rate of AIDS mortality that is based on a previously documented distribution of AIDS survival after infection (23). Bootstrapping (10,000 iterations) was used to generate 95% confidence intervals (CI) of incidence rates (24, 25).

### Ethics statement

Our study did not need an ethics committee approval because it relies entirely on published data made available by the Demographic and Health Surveys Program (13).

## Results

### Spatial association between male circumcision and HIV

**Figure 2:**
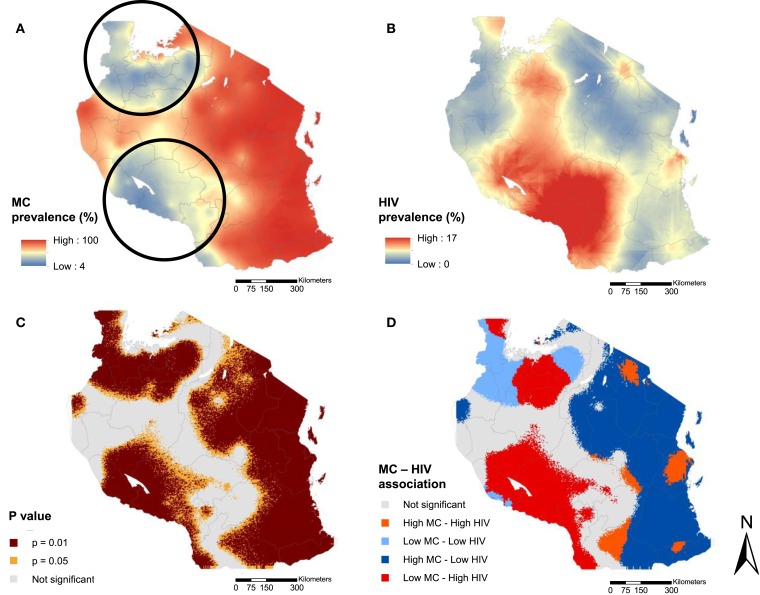
**Continuous surface maps of male circumcision (MC) prevalence (A) and HIV prevalence (B) in Tanzania estimated in the year 2012**. Black circles in **(A)** indicate the location of the MC cold spots identified by spatial scan statistics. Areas with significant association between MC and HIV prevalence are shown in **(C)**, and the type of the association is identified in **(D)**.

The continuous surface map of MC prevalence in Figure 1A illustrates the high MC prevalence in eastern Tanzania, where more than 90% of the male population was estimated to be circumcised. The map also highlights two discernible areas, one located in northern and the other in southwestern Tanzania, where MC prevalence is noticeably lower compared to the eastern region. These areas with low MC prevalence visually overlap with areas with high HIV prevalence (Figures 2A,B). The bivariate LISA analysis identified a statistically significant low MC–high HIV prevalence association in a northern region of the country, particularly in the districts of Mwanza, Shinyanga, and parts of the districts of Geita, Tabora, and Simiyu (Figure 1A). Similarly, a low MC–high HIV prevalence association was observed in a southwestern region of the country, particularly in the districts of Mbeya, Katavi, Rukwa, and Njombe (Figures 2C,D).

Furthermore, our analysis indicated a significantly high MC–low HIV prevalence association in an eastern region of the country, which as expected, had the highest MC prevalence. Notably, however, there was a high MC–high HIV prevalence association observed in the district of Dar es Salaam (Figure 2D) where, regardless of the high percentage of circumcised males, HIV prevalence was markedly higher compared to neighboring districts with similarly high MC prevalence.

### Spatial cold spots of male circumcision

Scan statistics identified two MC cold spots, one located in the northern districts of Shinyanga, Geita, Mwanza, Kagera, and Simiyu, and the other located mainly in the southwestern districts of Mbeya, Njombe, Rukwa, and Katavi (Figure 2A, dark circles). These MC cold spots were in locations where the bivariate LISA analysis indicated a significant low MC–high HIV prevalence association (Figures 2A,D). Moreover, the geographical distribution of the MC prevalence as well as the location of MC cold spots has remained almost the same over time (Figure S1 in Supplementary Material).

Table 1 summarizes epidemiological characteristics within and outside the MC cold spots. MC prevalence within the MC cold spots in 2004 was 41.83% (95% CI 39.04–44.63), and it declined, but not significantly, to 40.93% in 2012 (95% CI 38.81–43.04). The MC prevalence outside the MC cold spots in 2004 was 91.66% (95% CI 90.51–92.83), and it remained almost unchanged in 2012 (91.67%; 95% CI 90.69–92.65).

**Table 1:** **Epidemiological characteristics outside of male circumcision cold spots and within male circumcision cold spots**.

	2003–04		2007–08		2011–12	
	Outside MC cold spots (%)	Within MC cold spots (%)	Outside MC cold spots (%)	Within MC cold spots (%)	Outside MC cold spots (%)	Within MC cold spots (%)
	95% CI	95% CI	95% CI	95% CI	95% CI	95% CI
Total fraction of the population	58.71 (57.58–59.84)	41.29 (40.16–42.41)	56.63 (55.51–57.75)	43.37 (42.24–44.48)	60.71 (59.79–61.60)	39.29 (38.40–40.20)
Fraction of the male population	57.72 (55.99–59.45)	42.28 (40.56–44.01)	55.66 (53.92–57.40)	44.34 (42.59–46.08)	60.23 (58.84–61.60)	39.77 (38.39–41.16)
Fraction of the female population	59.46 (57.97–60.95)	40.54 (39.05–42.03)	57.30 (55.83–58.74)	42.70 (41.25–44.16)	61.04 (59.86–62.23)	38.96 (37.77–40.14)
Total HIV prevalence	6.88 (6.15–7.60)	9.02 (7.92–10.11)	5.32 (4.67–5.96)	8.45 (7.43–9.47)	4.36 (3.83–4.90)	6.41 (5.72–7.11)
HIV prevalence in the male population	5.70 (4.68–6.71)	8.44 (6.87–10.02)	3.88 (2.99–4.78)	7.35 (5.88–8.82)	2.77 (2.10–3.47)	5.48 (4.49–6.47)
HIV prevalence in the female population	7.74 (6.74–8.74)	9.46 (7.95–10.97)	6.24 (5.36–7.13)	9.22 (7.83–10.61)	5.49 (4.72–6.27)	7.12 (6.14–8.10)
Male circumcision prevalence	91.66 (90.51–92.83)	41.83 (39.04–44.63)	91.47 (90.27–92.67)	36.14 (33.46–38.81)	91.67 (90.69–92.65)	40.93 (38.81–43.04)
RR of HIV infection in females compared to males	1.25 (1.01–1.53)	1.17 (0.93–1.46)	1.53 (1.19–1.96)	1.12 (0.88–1.43)	1.78 (1.42–2.24)	1.03 (0.85–1.26)
RR of HIV infection in the male population	Ref	1.73 (1.36–2.20)	Ref	2.65 (1.99–3.5)	Ref	2.73 (2.12–3.53)
RR of infection in the female population	Ref	1.39 (1.16–1.67)	Ref	1.98 (1.63–2.40)	Ref	1.83 (1.54–2.16)

*MC, male circumcision; CI, confidence interval; RR, relative risk*.

The 2011–12 TDHS collected data on age at circumcision (13). According to these data, the median age (in years) at circumcision outside of the MC cold spots was 11 (range 41). Conversely, circumcision was practiced at older ages in males within the MC cold spots, with a median age at circumcision of 15 (range 42). Moreover, the age-specific MC prevalence was relatively stable across the TDHS rounds for all ages among males located both within (Figure S2A in Supplementary Material) and outside (Figure S2B in Supplementary Material) the MC cold spots.

**Figure 3:**
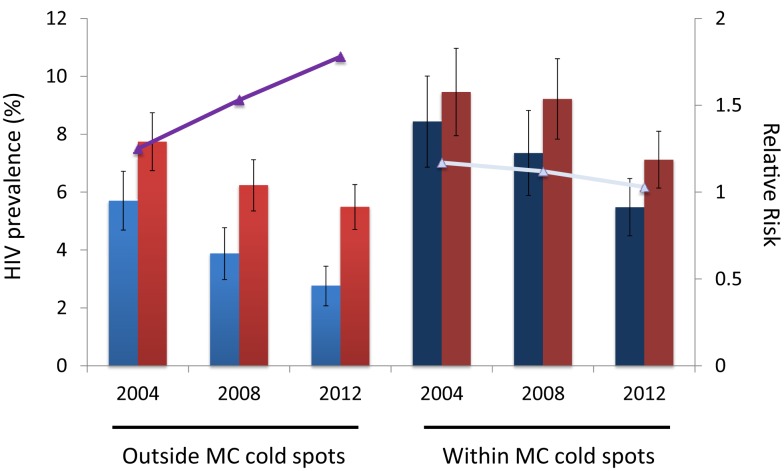
**HIV prevalence for males (blue bars) and females (red bars) outside and within the male circumcision cold spots**. Continuous lines (purple and gray) illustrate the relative risk of HIV infection in females compared to males.

The RR of HIV infection for males within the MC cold spots compared to males located outside the cold spots was 1.73 (95% CI 1.36–2.20) in 2004, and 2.73 (95% CI 2.12–3.53) in 2012 (Table 1). The RR of HIV infection for females within the cold spots compared to females located outside these areas was 1.39 (95% CI 1.16–1.67) in 2004, and 1.83 (95% CI 1.54–2.16) in 2012. The RR of infection in females compared to males outside the MC cold spots was 1.25 (95% CI 1.01–1.53) in 2004, and 1.78 (95% CI 1.42–2.24) in 2012 (Table 1). The RR of infection in females compared to males inside the MC cold spots was not statistically significant in 2004 or in 2012, with RR of 1.17 (95% CI 0.93–1.46) and 1.03 (95% CI 0.85–1.26), respectively (Figure 3).

### Geographical distribution of HIV incidence rate

**Figure 4:**
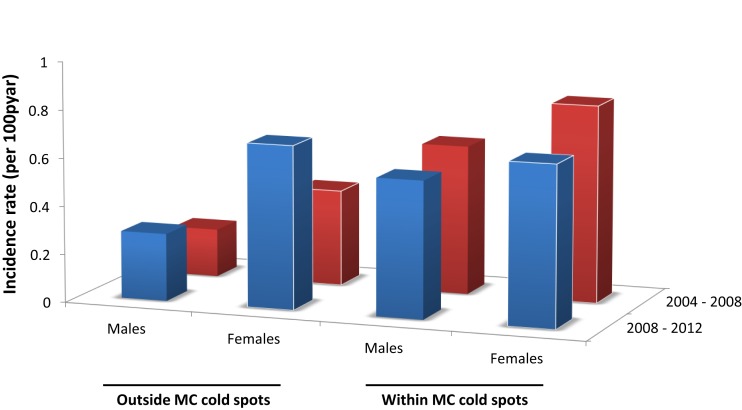
**HIV incidence rate for males and females outside and within the male circumcision cold spots during 2004–2008 (red bars) and 2008–2012 (blue bars)**.

HIV incidence rate for males outside the MC cold spots during the period 2004–08 was 0.20 per 100 person-years at risk (pyar) (95% CI 0.08–0.32), and slightly increased during the subsequent time period (2008–12) when it was 0.28/100 pyar (95% CI 0.18–0.40) (Figure 3). For females outside the MC cold spots, HIV incidence rate during 2004–08 was 0.40/100 pyar (95% CI 0.27–0.50), and it increased substantially during the next period (2008–12) to 0.68/100 pyar (95% CI 0.52–0.87) (Figure 4). HIV incidence rate within the MC cold spots for males was 0.62/100 pyar (95% CI 0.38–0.90) during the period 2004–08, and it slightly decreased during the subsequent time period to 0.57/100 pyar (95% CI 0.34–0.83). For females within the MC cold spots, HIV incidence rate during 2004–08 was 0.82/100 pyar (95% CI 0.58–1.11), and in contrast to the pattern observed for females outside the MC cold spots, HIV incidence rate during the next period (2008–12) decreased to 0.67/100 pyar (95% 0.44–0.91) (Figure 4).

## Discussion

Our study provides evidence for a geographical association between MC prevalence and HIV infection in Tanzania, and suggests that MC could be one of the factors driving the spatial distribution of the HIV epidemic in this country. We identified with statistical significance a spatial association between MC and HIV in areas with low percentage of circumcised males, and in which a large proportion of the HIV epidemic is concentrated. These areas are located in the northern and southwestern parts of the country between the districts of Shinyanga, Geita, Mwanza, Kagera, Simiyu, Mbeya, Njombe, Rukwa, and Katavi. Most of these districts have been targeted for VMMC scale-up since 2009 (9).

We propose that MC could be influencing the geographical distribution of the HIV epidemic in this country as well as the local dynamics of the infection. Current evidence suggests substantial declines in HIV prevalence in parts of West, Southern, and East Africa (9, 13). However, a recent study showed a striking spatial variability in the declines in HIV prevalence within some countries, including Tanzania (7). Aligned with this pattern, our study identified sharp declines in HIV prevalence in areas with existing low HIV prevalence and high MC prevalence, whereas areas with high HIV prevalence and low MC prevalence exhibited slower declines.

Our analyses suggest that besides affecting the geographical trends in HIV prevalence, MC could also be associated with different dynamics in HIV transmission among males and females. As expected, males located within the MC cold spots have a higher risk of HIV infection compared to males located outside these areas. However, the decline in HIV prevalence among males located outside the MC cold spots was much faster than the HIV prevalence decline in males within these areas (6). Therefore, the risk of infection in males within the MC cold spots compared to males outside these cold spots was increasing over time and was nearly three times higher by 2012.

A rapid decline in HIV prevalence in the male population located outside the MC cold spots could have also generated larger differences between HIV prevalence in males compared to females. We estimated that by 2012, females located outside the MC cold spots had twice the risk of infection compared to males. In contrast, the risk of infection in females compared to males located within the MC cold spots was approximately the same, and the RR remained stable over time.

Our findings suggest that the HIV infection burden in Tanzania could be concentrating in the female population in areas where most of the males are partially protected by MC. Despite the evidence that MC could generate indirect benefit to the female population by lowering the HIV prevalence in the male population (9), we calculated that for the last TDHS round (2011–12), the female population located outside the MC cold spots had almost 50% higher HIV prevalence compared to the male population, and that HIV incidence rate among females increased for the period 2008–12 compared to the previous period (2004–08). In contrast, HIV prevalence in the female population located within the MC cold spots was 23% higher than the male population, and HIV incidence rate among females was declining.

We estimated that HIV incidence rate in the female population located outside the MC cold spots has increased almost 40% between the time period 2004–08 and 2008–12. HIV incidence rate calculated for the female population located outside the MC cold spots in 2008–12 was fairly similar to the incidence rate for the female population within the MC cold spots, which at the same time was slightly higher to HIV incidence rate in the male population within MC cold spots during the same period. In contrast, HIV incidence rate in the male population outside MC cold spots was very low compared to the incidence rate estimated for the other populations, and it has been relatively stable during both periods.

It is important to note an exception in the spatial association between MC and HIV described above, which was observed in the Dar es Salaam region. Despite the high MC prevalence in this part of the country (almost 100% of the males are circumcised), HIV prevalence was also one of the highest in the country, with more than 7% of the population being infected with the virus. Consistent with the pattern observed in areas with high MC prevalence described above, the female population was suffering disproportional HIV infection burden. This region has the largest urban population in Tanzania. These urban populations are characterized by the predominance of high risk behaviors, which could be key factors for explaining the disproportionately high HIV prevalence in this part of the country, particularly in the female population (26–29). Further data and in-depth analysis is required to understand the epidemic status in this region.

Several study limitations could have affected our results. Given the multiple logistical difficulties in conducting the DHS, some of our measures could have been affected by inherent biases in the data, such as the variability in response rates to HIV testing and under-sampling of mobile individuals and key subpopulations at risk (30, 31). Moreover, the methodology used to calculate HIV incidence rate does not take into account migration or movement of individuals from one region to another within the country. Estimates of regional HIV incidence rate could be affected by patterns of migration. Anti-retroviral therapy coverage, and its impact on HIV survival, could also affect these incidence rate estimates. An additional potential bias is the global positioning system displacement process of the DHS sampling data points, used to preserve their confidentiality (32). This process could have an impact on the precision of the geographical location of the MC cold spots by a few kilometers. Lastly, since we analyzed data from cross-sectional surveys, we were not able to definitively establish the time ordering of HIV infection and MC circumcision. However, we estimated that the median age at circumcision was 11 years outside the MC cold spots and 15 years inside the MC cold spots; both ages suggest that circumcision predates sexual activity for the vast majority of the population. Therefore, circumcision after an individual becomes HIV infected is highly unlikely. Likewise, data for circumcised males were derived from self-reported circumcision status rather than physical examination. Some studies have reported the validity of self-reported MC data (33, 34), but others have found less satisfactory accuracy, partly due to differences in the amount of foreskin removed, and this may vary between populations or by ethnic group (35).

In sum, our results suggest that MC could be an important factor driving the geographical distribution of the HIV epidemic in Tanzania. Although HIV prevalence is declining in Tanzania, the decline was much slower in areas where MC prevalence is low compared to areas with high MC prevalence. Therefore, the ongoing scale-up of VMMC may have a considerable impact on the epidemic in Tanzania. Furthermore, the HIV infection burden could be concentrating in the female population in areas where most male individuals are partially protected by MC, and the risk of HIV infection for females located in these areas could be increasing over time. Therefore, along with VMMC, special efforts targeting the female population should be considered.

## Author Contributions

DC conceived the study and its design, conducted most of the statistical analyses, and wrote the first draft of the paper. LA-R contributed to study conception and design, conducting the statistical analyses, interpretation of the results, and writing of the manuscript.

## Conflict of Interest Statement

The authors declare that the research was conducted in the absence of any commercial or financial relationships that could be construed as a potential conflict of interest.

## Supplementary Material

The Supplementary Material for this article can be found online at http://journal.frontiersin.org/article/10.3389/fpubh.2015.00218

## Funding

The Qatar National Research Fund (JSREP 3-014-3-007), the Biostatistics, Epidemiology, and Biomathematics Research Core at the Weill Cornell Medical College – Qatar.

## Abbreviations

CI, confidence intervals; ELISA, enzyme-linked immunosorbent assay; MC, male circumcision; RR, relative risk; TDHS, Tanzania Demographic and Health Surveys; UNAIDS, Joint United Nations Programme on HIV/AIDS; VMMC, voluntary medical male circumcision; WHO, World Health Organization.
