# Supplementary material for: Are Geographical “Cold Spots” of Male Circumcision Driving Differential HIV Dynamics in Tanzania?
Source: Front Public Health. 2015 Sep 29;3:218. doi: 10.3389/fpubh.2015.00218 (PMC4586325; doi:10.3389/fpubh.2015.00218)
Supplement: Supplementary file 1 [file Image_1.PDF]

Supplementary figures

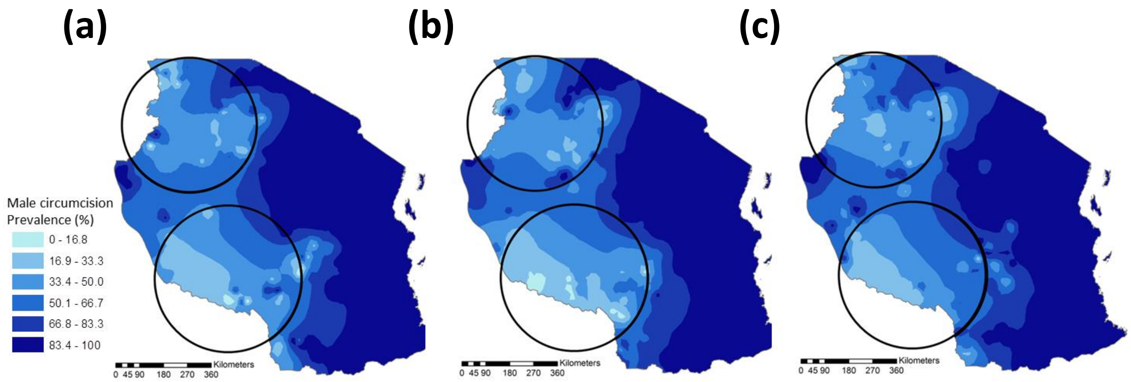

Figure S1. Continuous surface maps of male circumcision (MC) prevalence in (a) 2004, (b) 2008, (c) 2012. Black circles indicate the location of the MC cold spots identified by spatial scan statistics
