# Supplementary material for: Are Geographical “Cold Spots” of Male Circumcision Driving Differential HIV Dynamics in Tanzania?
Source: Front Public Health. 2015 Sep 29;3:218. doi: 10.3389/fpubh.2015.00218 (PMC4586325; doi:10.3389/fpubh.2015.00218)
Supplement: Supplementary file 2 [file Image_2.PDF]

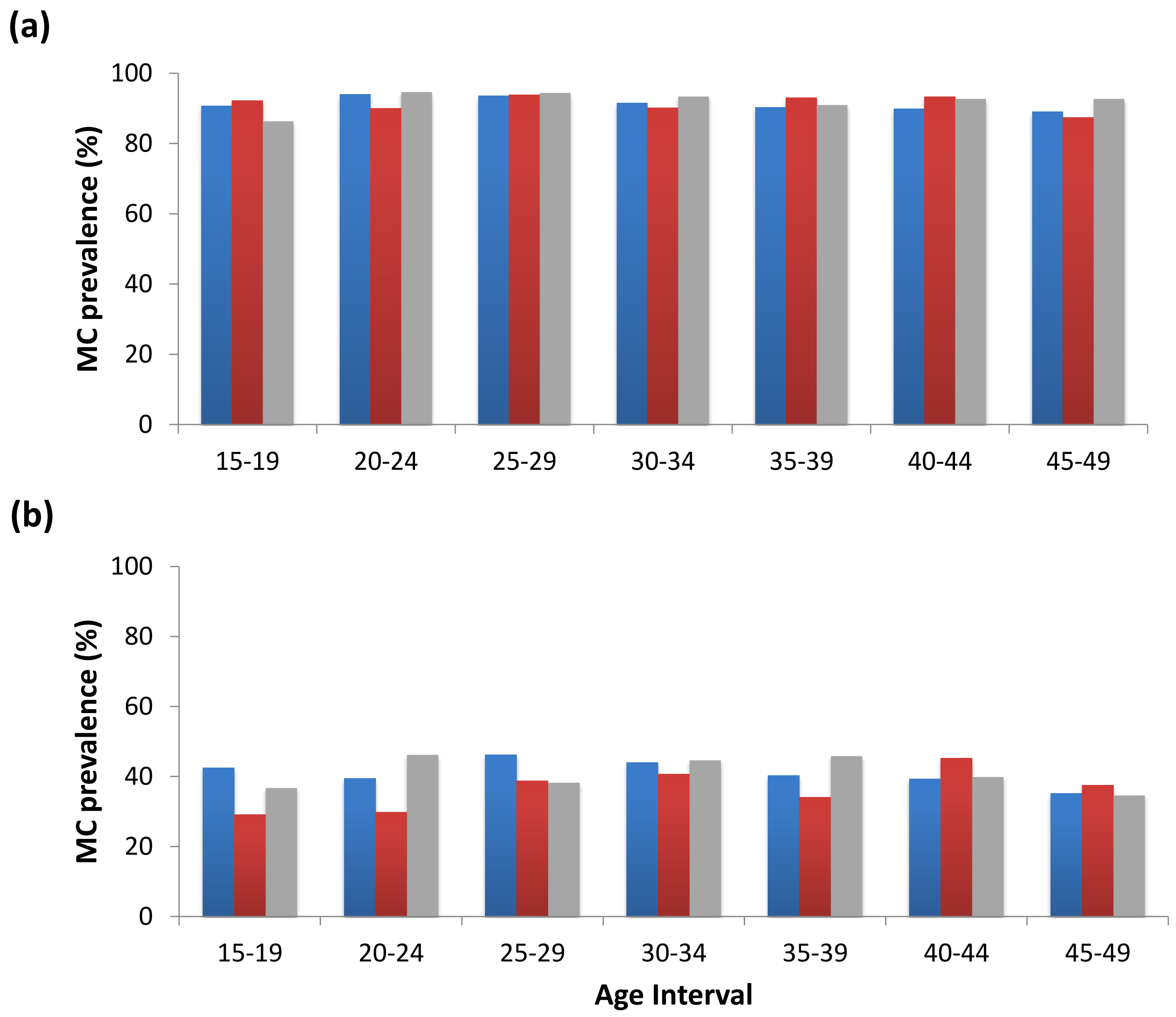

Figure S2. Age-specific (five-year age group) MC prevalence for males outside the male circumcision cold spots (a) and within the male circumcision cold spots (b) for the Demographic and Health Surveys conducted in Tanzania in 2003-04 (blue bars), 2007-08 (red bars), and 2011-12 (grey bars)
